# The impacts of thyroid function on the diagnostic accuracy of Cystatin C to detect acute kidney injury in ICU patients: a prospective, observational study

**DOI:** 10.1186/cc13186

**Published:** 2014-01-09

**Authors:** Feilong Wang, Wenzhi Pan, Hairong Wang, Yu Zhou, Shuyun Wang, Shuming Pan

**Affiliations:** 1 Department of Emergency, Xinhua Hospital Affiliated to Shanghai Jiaotong University School of Medcine, NO.1665, Kongjiang Road, Shanghai 200092, China; 2 Department of Cardiology, Shanghai Institute of Cardiovascular Diseases, Zhongshan Hospital of Fudan University, NO.180, Fenglin Road, Shanghai 200032, China

## Abstract

**Introduction:**

Cystatin C (Cysc) could be affected by thyroid function both in vivo and in vitro and thereby may have limited ability to reflect renal function. We aimed to assess the association between Cysc and thyroid hormones as well as the effect of thyroid function on the diagnostic accuracy of Cysc to detect acute kidney injury (AKI).

**Methods:**

A total of 446 consecutive intensive care unit (ICU) patients were screened for eligibility in this prospective AKI observational study. Serum Cysc, thyroid hormones and serum creatinine (Scr) were measured upon entry to the ICU. We also collected each patient's baseline characteristics including the Acute Physiology and Chronic Health Evaluation II (APACHE-II) score. The diagnostic performance of Cysc was assessed from the area under the receiver operator characteristic curve (AUC) in each quartile of thyroid hormone(s).

**Results:**

A total of 114 (25.6%) patients had a clinical diagnosis of AKI upon entry to the ICU. The range of free thyroxine (FT4) value was 4.77 to 39.57 pmol/L. Multivariate linear regression showed that age (standardized beta = 0.128, *P* < 0.0001), baseline Scr level (standardized beta = 0.290, *P* < 0.0001), current Scr (standardized beta = 0.453, *P* < 0.0001), albumin (standardized beta = -0.086, *P* = 0.006), and FT4 (standardized beta = 0.062, *P* = 0.039) were related with Cysc. Patients were divided into four quartiles based on FT4 levels. The AUC for Cysc in detecting AKI in each quartile were as follows: 0.712 in quartile I, 0.754 in quartile II, 0.829 in quartile III and 0.797 in quartile IV. There was no significant difference in the AUC between any two groups (all *P* > 0.05). The optimal cut-off value of Cysc for diagnosing AKI increased across FT4 quartiles (1.15 mg/L in quartile I, 1.15 mg/L in quartile II, 1.35 mg/L in quartile III and 1.45 mg/L in quartile IV).

**Conclusions:**

There was no significant impact of thyroid function on the diagnostic accuracy of Cysc to detect AKI in ICU patients. However, the optimal cut-off value of Cysc to detect AKI could be affected by thyroid function.

## Introduction

Acute kidney injury (AKI) is a prevalent problem and still a big challenge to both the developed and developing world [1]. About two-thirds of intensive care unit (ICU) patients develop an episode of AKI during their ICU stay [2]. Both short-term and long-term mortality were higher in ICU patients with AKI than those without [3-5]. Studies have found that early detection and treatment of AKI may improve outcomes [2]. Thus, timely diagnosis of AKI development after renal insult is urgent. Cystatin C (Cysc), a 13-kDa cysteine proteinase inhibitor, is freely filtered at the glomerulus and neither secreted nor reabsorbed by renal tubules. This physiological feature makes Cysc an ideal glomerular filtration biomarker. During the past few years, some studies have focused on the potential value of Cysc for the diagnosis and early detection of AKI [6-14]. However, these studies have reported conflicting results. Some studies reported good discrimination for Cysc in the early detection of AKI in various patient populations [7-9,14,15], while other studies found that Cysc had poor or moderate ability to predict AKI [6,10-13]. Besides that, there was no consensus about the appropriate cut-off value for using Cysc to diagnose or predict AKI [6-14,16]. These inconsistent results limit the usefulness of Cysc in the early detection of AKI in clinical practice.

Cysc is produced by all nucleated cells in the human body at a relatively constant rate [17]. However, recent studies found that thyroid hormones could stimulate the production of Cysc *in vitro*[18,19]. Moreover, clinical studies also found that Cysc was significantly associated with thyroid function [20-24]. The impact of thyroid hormones on the diagnostic value of Cysc in detecting AKI has raised concerns in clinical practice [25-27]. To the best of our knowledge, whether thyroid hormones are related to the level of Cysc in ICU patients has never been investigated. The effect of thyroid hormones on the diagnostic accuracy and threshold of Cysc in predicting AKI has also not been defined.

Therefore, we undertook a prospective, observational study in a large population of unselected ICU patients to assess: 1) the relationship between Cysc and thyroid hormones; and 2) the effect of thyroid function on the diagnostic value of Cysc in detecting AKI.

## Material and methods

### Participants

This prospective study recruited consecutive patients 18 years old and older hospitalized in the ICU of Xinhua Hospital affiliated with Shanghai Jiaotong University School of Medicine between April 2011 and May 2012, including medical and trauma patients. We decided *a priori* to exclude patients according to the following criteria: 1) past history of any thyroid diseases, such as hyperthyroidism, hypothyroidism and thyroid tumors; 2) thyroid nodule found by physical examination when admitted to ICU; 3) pregnancy within the previous six months; 4) undergoing any hormone replacement therapy except insulin use; 5) pre-existing severe renal disease (serum creatinine (Scr) >300 umol/L) or pre-existing dialysis; and 6) undergoing continuous renal replacement therapy (CRRT) in the four weeks before the blood sample was collected. Patients who died or were discharged from the ICU within four hours of admission were also excluded because data collection was difficult for these patients. The Shanghai Jiaotong University Xinhua Hospital Ethics Committee approved the study and waived the requirement for informed consent, because this was an observational study and all laboratory indices observed were commonly measured for all patients in our ICU department.

### Definition of acute kidney injury

The patients were diagnosed as having AKI by using the stage 1 AKI criteria of the Acute Kidney Injury Network (AKIN) classification: new-onset of at least 1.5-fold increase or ≥0.3 mg/dL (26.5 umol/L) increment of SCr from baseline. If the preadmission Scr was available, we defined baseline creatinine by using the following rules ranked in descending order of preference: 1) the lowest value between three and 365 days preadmission; 2) the lower value of either preadmission Scr within three days preadmission or the lowest Scr measurement in seven days after admission to the ICU, if it was less than the initial creatinine on entry to the ICU; and 3) the lowest value of preadmission Scr within three days. If the value of 2) and 3) were greater than the initial creatinine on entry to the ICU or if the preadmission value was unavailable, then the lower value of either the initial creatinine on entry to the ICU or the lowest pCr measurement in seven days was used. We did not use the Modification of Diet in Renal Disease (MDRD) formula to back-calculate baseline creatinine because using this method to estimate baseline creatinine would result in an overestimation of the prevalence of AKI [28].

### Clinical data and laboratory methods

We prospectively collected each patient's demographic and clinical characteristics, including the Acute Physiolgy and Chronic Health Evaluation II (APACHE-II) score (which can range from 0 to 71, with higher scores indicating more severe illness). Serum Cysc, creatinine, glucose, lactate, electrolytes and albumin levels were measured using a Hitachi 7600–120 analyzer (Hitachi, Tokyo, Japan). The analytical range of Cysc in the laboratory of our hospital is 0.4 to 7.5 mg/L. The reported total coefficients of variation are 4.2% at mean concentrations 0.6 mg/L and 3.8% at 1.4 mg/L, respectively. Thyroid hormones, including free triiodothyronine (FT3), free thyroxine (FT4), total triiodothyronine (TT3), total thyroxine (TT4) and thyroid-stimulating hormone (TSH), were measured using the ADVIA Centaur immunoassay system (Siemens Healthcare Diagnostics Inc, Tarrytown, NY, USA). Reverse triiodothyronine (rT3) level was measured using the Maglumi 1000 Analyzer chemiluminescence immunoassay system (SNIBE Co, Ltd, Guandong, China). Serum C-reactive protein (CRP) levels were measured using the QuikRead CRP test kit (Orion Diagnostica, Espoo, Finland). We calculated the estimated glomerular filtration rate (eGFR) by using the abbreviated MDRD study equation. Blood samples were collected from all eligible patients when they were admitted to the ICU.

### Statistical analysis

Continuous variable and categorical variables were presented as mean value ± SD and precent, respectively. Bivariate correlation analysis was utilized to examine the association between two variables. Multivariate linear regression was performed to search for factors independently associated with Cysc. A criterion of *P* <0.05 for entry and *P* ≥0.10 for removal was imposed in this procedure. We divided FT4 into quartiles and compared demographics, clinical characteristics and laboratory test results with analysis of variance for continuous variables and chi-square or Fisher’s exact tests for categorical variables. Receiver operator characteristic (ROC) curve was used to examine the performance of Cysc to detect AKI. The curve represented a plot of sensitivity versus 1-specificity. The area under the curve (AUC) was derived from the ROC curve. The differences between AUC were tested by Hanley – McNeil methods [29]. A statistically derived value, based on the Youden index16, maximizing the sum of the sensitivity and specificity was used to define the optimal cut-off value. A two-sided *P* value <0.05 was considered to indicate statistical significance. All analyses were performed with SPSS 13.0 software.

## Results

### Base-line characteristics and base-line factors related to Cysc

Cysc and thyroid hormones were missed in 12 patients. Those patients were excluded from the study. A total of 446 patients were screened for eligibility in this analysis. The mean age was 69.07 ± 15.58 years and 58.3% were men. Mean Apache-II score was 15.46 ± 7.34. On entry to the ICU, 114 (25.6%) had a clinical diagnosis of AKI. A total of 56 patients died during their ICU stay. In Table 1, bivariate correlation analysis shows that a higher level of Cysc was associated with older age, higher level of baseline Scr and current Scr, higher Apache-II score and lower level of albumin. In addition, there was a negative association between Cysc and TT3 as well as FT3, and a positive association between Cysc and FT4. In multivariate linear regression (Table 2), age (standardized beta = 0.128, *P* <0.0001), baseline Scr level (standardized beta = 0.290, *P* <0.0001), current Scr (standardized beta = 0.453, *P* <0.0001), albumin (standardized beta = -0.086, *P* = 0.006), and FT4 (standardized beta = 0.062, *P* = 0.039) were related to Cysc.

**Table 1:** Factors associated with Cysc by bivariate correlation analysis

**Variables**	**Cysc**	
	**R**	***P***
Current Scr (umol/L)	0.684	0.000
Baseline Scr (umol/L)	0.562	0.000
Sex	0.027	0.568
Age (years)	0.380	0.000
Albumin (g/L)	-0.230	0.000
TT3 (nmol/L)	-0.099	0.036
TT4 (nmol/L)	0.005	0.916
FT3 (pmol/L)	-0.180	0.000
FT4 (pmol/L)	0.098	0.039
TSH (IU/mL)	0.168	0.054
rT3 (nmol/L)	-0.005	0.911
APACHE-II score	0.429	0.000

APACHE II score, Acute Physiology and Chronic Health Evaluation II score; Baseline Scr, baseline serum creatinine; Current Scr, serum creatinine on the entry to ICU; FT3, free triiodothyronine; FT4, free thyroxine; rT3, reverse triiodothyronine; TSH, thyroid-stimulating-hormone; TT3, total triiodothyronine; TT4, total thyroxine.

**Table 2:** Factors associated with Cysc in multivariate linear regression

**Independent variables**^**a**^	**Cysc**	
	**Standardized β**	***P***
Current Scr (umol/L)	0.486	0.000
Baseline Scr (umol/L)	0.290	0.000
Age (years)	0.128	0.000
Albumin (g/L)	-0.086	0.006
FT4 (pmol/L)	0.062	0.039
Constant	0.291 (Unstandardized)	0.109

^a^Independent variables included age, sex, baseline Scr, current Scr, albumin, TT3, TT4, FT3, FT4, TSH. Variables not listed in the table were removed from the stepwise analysis. Adjusted R square 0.627. Baseline Scr, baseline serum creatinine; Current Scr, serum creatinine on the entry to ICU; FT3, free triiodothyronine; FT4, free thyroxine; rT3, reverse triiodothyronine; TSH, thyroid-stimulating-hormone; TT3, total triiodothyronine; TT4, total thyroxine.

Differences in clinical and laboratory characteristics among the quartiles of FT4 are listed in Table 3. Patients in the higher quartiles of FT4 tended to be older and had higher levels of albumin, TT4 and FT3, and a low level of TSH. However, there was no significant difference among the four quartiles in other clinical and laboratory characteristics, including sex, level of baseline Scr, current Scr, Cysc, TT3, rT3, APACHE-II score as well as the prevalence of AKI.

**Table 3:** Baseline clinical and laboratory characteristics by quartile of FT4

	**I**	**II**	**III**	**IV**	***P***
Number	111	111	112	112	/
Age (years)	65.2 ± 16.7	69.4 ± 15.6	71.3 ± 15.1	70.3 ± 14.4	0.020
Men, Number (%)	65 (58.6)	67 (60.4)	72 (64.3)	56 (50.0)	0.171
APACHE-II score (points)	15.6 ± 7.3	15.1 ± 7.9	14.9 ± 6.7	16.3 ± 7.4	0.524
Baseline Scr (umol/L)	80.0 ± 45.9	80.0 ± 39.0	86.6 ± 46.4	84.0 ± 48.7	0.636
Current Scr (umol/L)	98.2 ± 89.4	100.7 ± 61.5	108.1 ± 79.7	106.8 ± 76.1	0.741
Cysc (mg/L)	1.23 ± 0.65	1.23 ± 0.51	1.32 ± 0.67	1.35 ± 0.79	0.354
Albumin (g/L)	32.7 ± 4.7	35.0 ± 6.1	35.1 ± 5.0	35.4 ± 5.5	0.000
TT3 (nmol/L)	0.83 ± 0.39	0.94 ± 0.34	0.89 ± 0.29	0.90 ± 0.36	0.109
TT4 (nmol/L)	72.04 ± 28.93	89.17 ± 23.00	90.03 ± 20.98	94.32 ± 24.29	0.000
FT3 (pmol/L)	3.04 ± 0.62	3.27 ± 0.54	3.42 ± 0.59	3.66 ± 0.92	0.000
FT4 (pmol/L)	11.93 ± 1.54	14.47 ± 0.53	16.14 ± 0.50	19.58 ± 3.17	0.000
TSH (IU/mL)	3.87 ± 16.16	1.26 ± 1.03	1.70 ± 1.84	1.14 ± 1.05	0.044
rT3 (nmol/L)	0.49 ± 0.21	0.54 ± 0.21	0.54 ± 0.24	0.57 ± 0.27	0.113
AKI (Number, %)	24 (21.6)	26 (23.4)	36 (32.1)	28 (25.0)	0.292

Quartile cut points for FT4 were 13.49, 15.38 and 17.07 pmol/L, continuous variables were compared with one-way ANOVA, and dichotomous variables were compared with the chi-square test. ANOVA, analysis of variance; APACHE II score, Acute Physiology and Chronic Health Evaluation II score; Baseline Scr, baseline serum creatinine; Current Scr, serum creatinine on the entry to ICU; Cysc, Cystatin C; FT3, free triiodothyronine; FT4, free thyroxine; rT3, reverse triiodothyronine; TSH, thyroid-stimulating-hormone; TT3, total triiodothyronine; TT4, total thyroxine.

### Association between thyroid hormones and APACHE-II scores

Among the thyroid hormones, only FT4 was not related to APACHE-II scores (*P* = 0.219). FT3 (r = -0.363, *P* <0.001), TT3 (r = -0.138, *P* <0.001), TT4 (r = -0.338, *P* <0.001), TSH (r = -0.133, *P* = 0.005) and rT3 (r = -0.205, *P* <0.001) were all correlated with APACHE-II scores.

### Association between Cysc and AKI in patients with different levels of FT4

**Figure 1:**
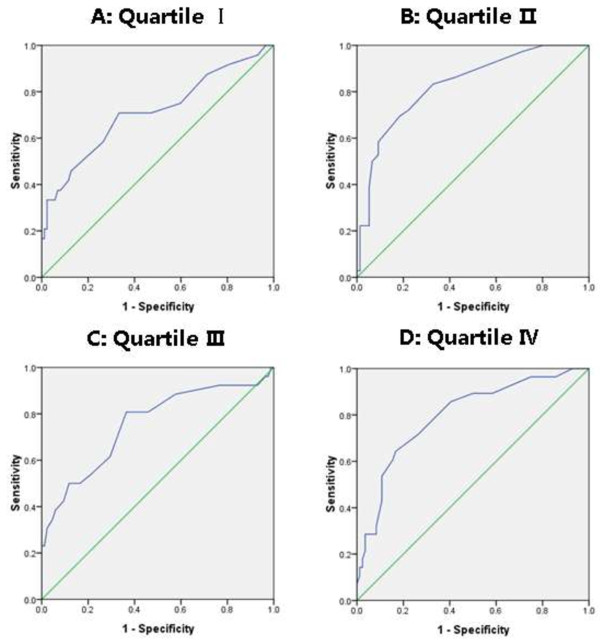
Receiver operating characteristic curves for Cysc in quartile I (A), quartile II (B), quartile III (C) and quartile IV (D).

To evaluate the impact of FT4 on the accuracy of Cysc in the diagnosis of AKI, a ROC curve was drawn in each quartile of FT4 (Figure 1). The AUC was calculated as 0.712 in quartile I, 0.754 in quartile II, 0.829 in quartile III and 0.797 in quartile IV (Table 4). There was no significant difference in AUC between any two groups. The optimal cut-off value of Cysc for diagnosing AKI increased across FT4 quartiles (quartile I and quartile II = 1.15 mg/L, quartile III =1.35 mg/L, quartile IV =1.45 mg/L).

**Table 4:** Performance of Cysc in diagnosing AKI by quartile of FT4

	**AUC ROC**	**95% CI**	**Cut-off**	**Sensitivity**	**Specificity**
I	0.712 ± 0.066	0.582-0.841	1.15	0.708	0.667
II	0.754 ± 0.059	0.638-0.870	1.15	0.808	0.635
III	0.829 ± 0.041	0.748-0.910	1.35	0.694	0.816
IV	0.797 ± 0.049	0.700-0.893	1.45	0.643	0.833

Quartile I versus Quartile II Z = 0.474, *P* = 0.318; Quartile I versus Quartile III Z = 1.506, *P* = 0.066; Quartile I versus Quartile IV Z = 1.034, *P* = 0.151; Quartile II versus Quartile III Z = 1.044, *P* = 0.148; Quartile II versus Quartile IV Z = 0.561, *P* = 0.288]. Quartile III versus Quartile IV Z = 0.501, *P* = 0.308. AUC ROC, area under the receiver operating characteristic curve; CI, confidence interval.

## Discussion

In this prospective study, we first assessed the impact of thyroid function on the accuracy of Cysc in the diagnosis of AKI in ICU patients. The primary findings of this study are as follows: (1) FT4 was the only index among thyroid hormones that was not correlated with APACHE-II score and still related to the Cysc level after adjusting for several confounding factors; (2) there was no significant difference in the diagnostic performance of Cysc among patients with different FT4 levels; and (3) the optimal cut-off value of Cysc in diagnosing AKI increased as FT4 increased.

Previous studies have shown that some thyroid hormones might be confounded by critical illness and a low FT3 level was correlated with the severity of morbidity and the outcomes of patients in ICUs [30,31]. Our present study also confirmed the negative association between FT3 level and APACHE-II score. As we know, the severity of illness score was positively associated with the risk of AKI as well as the level of Cysc, and thus FT3 was inversely related to Cysc. However, the level of FT4 was the only index among thyroid hormones that was not related to the APACHE-II score in this study. This indicated that FT4 was less affected by the severity of illness and could reflect a stable state of thyroid hormones as compared with other indices of thyroid hormones. So, FT4 can directly reflect the stimulating effect of thyroid hormones on Cysc and FT4 was positively related to Cysc. In fact, FT4 was the only index independently correlated with Cysc. Based on these results, we chose FT4 as the representative variable to assess the impact of thyroid function on the accuracy of Cysc in the diagnosis of AKI in ICU patients.

Cysc used to be considered less subject to the non-renal variables that impact Scr. However, more recent studies [18-24,32] have found that several factors are associated with higher Cysc in noncritically ill patients, including older age, male sex, current smoking status, elevated CRP levels as well as thyroid hormones. However, the factors associated with the Cysc level were not well defined in critically ill patients. In a study of 85 ICU patients, the investigator reported that the Cysc level was not affected by low T3 or T3/T4 syndrome [6]. In contrast with this study, we found that Cysc level was not only associated with both baseline Scr and current Scr levels, but also is related to age, and levels of albumin and FT4 in ICU patients.

Results relating to the diagnostic accuracy of Cysc in detecting AKI have been conflicting in previous studies. The AUC for Cysc ranged from 0.55 to 0.99 across different studies [16]. Some previous studies have found that thyroid hormones were associated with Cysc level both *in vitro*[18,19] and *in vivo*[20-24], but whether the thyroid function could affect the performance of Cysc to identify AKI was never investigated before. Our present study showed that the difference of AUC among different FT4 quartiles did not reach statistical significance. Although thyroid hormones could stimulate the production of Cysc at baseline as well as the progress of AKI after a critical illness, the acute decrease of GFR during critical illness was the most important factor for the increment of Cysc from baseline. This may be the reason. However, the optimal cut-off value of Cysc in the diagnosis of AKI increased with increasing FT4 quartile is an interesting finding. This result indicates that FT4 might stimulate Cysc production and, thus, affect the absolute value of Cysc and the optimal cut-off value of Cysc in the diagnosis of AKI. This result may be one of the explanations fpr a wide range of optimal cut-off values of Cysc in detecting AKI in previous studies [16]. It also should be taken into consideration when using Cysc to identify AKI in clinical settings.

Several limitations to our study exist. First, we used Cysc to diagnose rather than predict AKI. We could not exclude the possibility that some non-AKI patients progressed to AKI after their entry into the ICU. However, whether Cysc could serve as an early biomarker of AKI remains uncertain. Second, because some unknown non-GFR determinants of Cysc may exist, the relationship between Cysc and thyroid hormones would be changed if adjusting for those confounding factors.

## Conclusions

In this large-scale study of unselected ICU patients, we found that FT4 was the only index among thyroid hormones that was not correlated with APACHE-II score and still related to Cysc level after adjusting for several confounding factors. Thyroid function had no significant impact on the diagnostic accuracy of Cysc to detect AKI in ICU patients. However, the optimal cut-off value of Cysc to detect AKI could be affected by thyroid function.

## Key messages

• FT4 was the only index among thyroid hormones that was not correlated with APACHE-II score and still related to Cysc level after adjusting for several confounding factors.

• There was no significant impact of thyroid function on the diagnostic accuracy of Cysc to detect AKI in ICU patients.

• The optimal cut-off value of Cysc to detect AKI could be affected by thyroid function.

## Abbreviations

AKI: acute kidney injury; APACHE-II score: Acute Physiology and Chronic Health Evaluation II score; AUC: area under the curve; Cysc: Cystatin C; eGFR: estimated glomerular filtration rate; FT3: free triiodothyronine; FT4: free thyroxine; GFR: glomerular filtration rate; NTIS: nonthyroidal illness syndrome; ROC: receiver operator characteristic; rT3: reverse triiodothyronine; TSH: thyroid-stimulating hormone; TT3: total triiodothyronine; TT4: total thyroxine.

## Competing interests

The authors declare that they have no competing interests.

## Authors’ contributions

FW and SP contributed to the design, data collection and manuscript writing. WP and YZ contributed to the design and statistical analysis. HW and SW participated in data collection. All authors read and approved the final manuscript.

## References

[B1] Uchino S Kellum JA Bellomo R Doig GS Morimatsu H Morgera S Schetz M Tan I Bouman C Macedo E Gibney N Tolwani A Ronco C Beginning and Ending Supportive Therapy for the Kidney (BEST Kidney) investigators Acute renal failure in critically ill patients: a multinational, multicenter study JAMA 2005 294 813 818 10.1001/jama.294.7.813 16106006

[B2] Hoste EA Clermont G Kersten A Venkataraman R Angus DC De Bacquer D Kellum JA RIFLE criteria for acute kidney injury are associated with hospital mortality in critically ill patients: a cohort analysis Crit Care 2006 10 R73 R83 10.1186/cc4915 16696865 PMC1550961

[B3] Bagshaw SM George C Dinu I Bellomo R A multi-center evaluation of the RIFLE criteria for early acute kidney injury in critically ill patients Nephrol Dial Transplant 2008 23 1203 1210 17962378 10.1093/ndt/gfm744

[B4] Coca SG Yusuf B Shlipak MG Garg AX Parikh CR Long-term risk of mortality and other adverse outcomes after acute kidney injury: a systematic review and meta-analysis Am J Kidney Dis 2009 53 961 973 10.1053/j.ajkd.2008.11.034 19346042 PMC2726041

[B5] Li Q Fang JY Wang WP Liu JH Wang KK Cystatin C and serum creatinine in estimating acute kidney injury of shock patients World J Emerg Med 2010 1 185 189 25214965 PMC4129687

[B6] Herget-Rosenthal S Marggraf G Hüsing J Göring F Pietruck F Janssen O Philipp T Kribben A Early detection of acute renal failure by serum cystatin C Kidney Int 2004 66 1115 1122 10.1111/j.1523-1755.2004.00861.x 15327406

[B7] Che M Xie B Xue S Dai H Qian J Ni Z Axelsson J Yan Y Clinical usefulness of novel biomarkers for the detection of acute kidney injury following elective cardiac surgery Nephron Clin Pract 2010 115 c66 c72 10.1159/000286352 20173352

[B8] Chung MY Jun DW Sung SA Diagnostic value of cystatin C for predicting acute kidney injury in patients with liver cirrhosis Korean J Hepatol 2010 16 301 307 10.3350/kjhep.2010.16.3.301 20924213 PMC3304597

[B9] Haase M Bellomo R Devarajan P Ma Q Bennett MR Möckel M Matalanis G Dragun D Haase-Fielitz A Novel biomarkers early predict the severity of acute kidney injury after cardiac surgery in adults Ann Thorac Surg 2009 88 124 130 10.1016/j.athoracsur.2009.04.023 19559209

[B10] Ling Q Xu X Li JJ Chen J Shen JW Zheng SS Alternative definition of acute kidney injury following liver transplantation: based on serum creatinine and cystatin C levels Transplant Proc 2007 39 3257 3260 10.1016/j.transproceed.2007.03.107 18089366

[B11] Soto K Coelho S Rodrigues B Martins H Frade F Lopes S Cunha L Papoila AL Devarajan P Cystatin C as a marker of acute kidney injury in the emergency department Clin J Am Soc Nephrol 2010 5 1745 1754 10.2215/CJN.00690110 20576828 PMC2974372

[B12] Briguori C Visconti G Rivera NV Focaccio A Golia B Giannone R Castaldo D De Micco F Ricciardelli B Colombo A Cystatin C and contrast-induced acute kidney injury Circulation 2010 121 2117 2122 10.1161/CIRCULATIONAHA.109.919639 20439784

[B13] Liang XL Shi W Liu SX Yan LJ Xuan HJ Xiong WP Peng YQ Huang JS Liang YZ Prospective study of cystatin C for diagnosis of acute kidney injury after cardiac surgery Nan Fang Yi Ke Da Xue Xue Bao 2008 28 2154 2156 19114344

[B14] Nejat M Pickering JW Walker RJ Endre ZH Rapid detection of acute kidney injury by plasma cystatin C in the intensive care unit Nephrol Dial Transplant 2010 25 3283 3289 10.1093/ndt/gfq176 20350927

[B15] Royakkers AA Korevaar JC van Suijlen JD Hofstra LS Kuiper MA Spronk PE Schultz MJ Bouman CS Serum and urine cystatin C are poor biomarkers for acute kidney injury and renal replacement therapy Intensive Care Med 2011 37 493 501 10.1007/s00134-010-2087-y 21153403 PMC3042095

[B16] Zhang Z Lu B Sheng X Jin N Cystatin C in prediction of acute kidney injury: a systemic review and meta-analysis Am J Kidney Dis 2011 58 356 365 10.1053/j.ajkd.2011.02.389 21601330

[B17] Abrahamson M Olafsson I Palsdottir A Ulvsbäck M Lundwall A Jensson O Grubb A Structure and expression of the human cystatin C gene Biochem J 1990 268 287 294 2363674 10.1042/bj2680287 PMC1131430

[B18] Schmid C Ghirlanda-Keller C Zwimpfer C Zoidis E Triiodothyronine stimulates cystatin C production in bone cells Biochem Biophys Res Commun 2012 419 425 430 10.1016/j.bbrc.2012.02.040 22360852

[B19] Kotajima N Yanagawa Y Aoki T Tsunekawa K Morimura T Ogiwara T Nara M Murakami M Influence of thyroid hormones and transforming growth factor-β1 on cystatin C concentrations J Int Med Res 2010 38 1365 1373 10.1177/147323001003800418 20926009

[B20] Fricker M Wiesli P Brändle M Schwegler B Schmid C Impact of thyroid dysfunction on serum cystatin C Kidney Int 1944–1947 2003 63 10.1046/j.1523-1755.2003.00925.x 12675875

[B21] Wiesli P Schwegler B Spinas GA Schmid C Serum cystatin C is sensitive to small changes in thyroid function Clin Chim Acta 2003 338 87 90 10.1016/j.cccn.2003.07.022 14637271

[B22] Manetti L Pardini E Genovesi M Campomori A Grasso L Morselli LL Lupi I Pellegrini G Bartalena L Bogazzi F Martino E Thyroid function differently affects serum cystatin C and creatinine concentrations J Endocrinol Invest 2005 28 346 349 15966508 10.1007/BF03347201

[B23] Goede DL Wiesli P Brandle M Bestmann L Bernays RL Zwimpfer C Schmid C Effects of thyroxine replacement on serum creatinine and cystatin C in patients with primary and central hypothyroidism Swiss Med Wkly 2009 139 339 344 19529992 10.4414/smw.2009.12654

[B24] Ozden TA Tekerek H Baş F Darendeliler F Effect of hypo-and euthyroid status on serum cystatin C levels J Clin Res Pediatr Endocrinol 2010 2 155 158 10.4274/jcrpe.v2i4.155 21274315 PMC3005688

[B25] Bagshaw SM Bellomo R Cystatin C in acute kidney injury Curr Opin Crit Care 2010 16 533 539 10.1097/MCC.0b013e32833e8412 20736828

[B26] Soni SS Pophale R Ronco C New biomarkers for acute renal injury Clin Chem Lab Med 2011 49 1257 1263 21726165 10.1515/CCLM.2011.664

[B27] McIlroy DR Wagener G Lee HT Biomarkers of acute kidney injury: an evolving domain Anesthesiology 2010 112 998 1004 10.1097/ALN.0b013e3181cded3f 20216399

[B28] Pickering J Endre ZH Back-calculating baseline creatinine with MDRD misclassifies acute kidney injury in the intensive care unit Clin J Am Soc Nephrol 2010 5 1165 1173 10.2215/CJN.08531109 20498242 PMC2893073

[B29] Hanley JA McNeil BJ A method of comparing the areas under ROC curves derived from same cases Radiology 1983 148 839 843 6878708 10.1148/radiology.148.3.6878708

[B30] Wang F Pan W Wang H Wang S Pan S Ge J Relationship between thyroid function and ICU mortality: a prospective observation study Crit Care 2012 16 R11 10.1186/cc11151 22257427 PMC3396242

[B31] Economidou F Douka E Tzanela M Nanas S Kotanidou A Thyroid function during critical illness Hormones (Athens) 2011 10 117 124 21724536 10.14310/horm.2002.1301

[B32] Knight EL Verhave JC Spiegelman D Hillege HL de Zeeuw D Curhan GC de Jong PE Factors influencing serum cystatin C levels other than renal function and the impact on renal function measurement Kidney Int 2004 65 1416 1421 10.1111/j.1523-1755.2004.00517.x 15086483

